# Gentisaldehyde and Its Derivative 2,3-Dihydroxybenzaldehyde Show Antimicrobial Activities Against Bovine Mastitis *Staphylococcus aureus*

**DOI:** 10.3389/fvets.2018.00148

**Published:** 2018-07-11

**Authors:** Andrea Schabauer, Christoph Zutz, Barbara Lung, Martin Wagner, Kathrin Rychli

**Affiliations:** ^1^Institute of Milk Hygiene, University of Veterinary Medicine Vienna, Vienna, Austria; ^2^Bioactive Microbial Metabolites (BiMM), Bio-Resources & Technologies Tulln, Tulln, Austria; ^3^Veterinary Health Service Laboratory, Ried im Innkreis, Austria

**Keywords:** mastitis, 2, 3-dihydroxybenzaldehyde, 2, 5-dihydroxybenzaldehyde, gentisaldehyde, *Staphylococcus aureus*, antiseptic, antibiotic resistance

## Abstract

Bovine mastitis is a worldwide disease of dairy cattle associated with significant economic losses for the dairy industry. One of the most common pathogens responsible for mastitis is *Staphylococcus* (*S*.) *aureus*. Due to the development and spreading of antibiotic resistance, the search for novel antimicrobial substances against *S. aureus* is of great importance. The aim of this study was to evaluate two dihydroxybenzaldehydes for the prevention of bovine mastitis. Therefore we determined the minimal inhibitory concentration (MICs) of gentisaldehyde (2,5-dihydroxybenzaldehyde) and 2,3-dihydroxybenzaldehyde of a diverse set of 172 bovine mastitis *S. aureus* isolates using an automated robot-based microdilution method. To characterize the bovine isolates we determined the genotype by *spa*-typing, the antimicrobial resistance to eight antibiotic classes using the disk diffusion method and the MICs of three commonly used antiseptics (benzalkonium chloride, chlorhexidine, and iodine). Further we investigated the cytotoxicity of gentisaldehyde and 2,3-dihydroxybenzaldehyde in bovine mammary epithelial MAC-T cells using the XTT assay. The *S. aureus* strains showed a high genetic diversity with 52 different *spa*-types, including five novel types. Antibiotic susceptibility testing revealed that 24% of isolates were resistant to one antimicrobial agent and 3% of isolates were multi-resistant. The occurrence of antibiotic resistance strongly correlated with the *spa*-type. Both dihydroxybenzaldehydes showed antimicrobial activities with a MIC_50_ of 500 mg/L. The MIC of gentisaldehyde significantly correlated with that of 2,3-dihydroxybenzaldehyde, whereas no correlation was observed with the MIC of the three antiseptics. Cytotoxicity testing using bovine mammary epithelial MAC-T cells revealed that gentisaldehyde and 2,3-dihydroxybenzaldehyde show low toxicity at MIC_50_ and MIC_90_ concentrations. In conclusion, gentisaldehyde and 2,3-dihydroxybenzaldehyde exhibited antimicrobial activities against a diverse range of bovine mastitis *S. aureus* strains at low-cytotoxic concentrations. Therefore, both compounds are potential candidates as antiseptics to prevent bovine mastitis and to reduce the use of antibiotics in dairy cows.

## Introduction

Mastitis is an inflammatory disease of the mammary gland that is usually caused by bacterial infection (1). Bovine mastitis is today a worldwide problem in dairy cows leading to significant economic losses due to decreased milk yield. Although the lethality rate of cows suffering from mastitis is low, they have a higher risk for premature culling (2). Additionally, in the case of severe clinical mastitis, animals experience pain and discomfort leading to depressed appearance, weight loss, and abnormal postures (3). The most common mastitis pathogens include Gram-negative bacteria such as *Escherichia coli, Pseudomonas* spp. and *Klebsiella* spp. and the Gram-positive species *Staphylococcus* (*S*.) *aureus, Streptococcus agalactiae, Streptococcus dysgalactiae*, and *Streptococcus uberis* (1). *S. aureus* is known to be the primary agent responsible for bovine mastitis, including clinical as well as subclinical mastitis. Its special abilities to form biofilms or hide within host phagocytes and epithelial cells of the mammary gland might account for its ability to evade antibiotics, leading to prolonged therapy or even persistence (4).

Antimicrobial drug classes commonly used in mastitis therapy are beta-lactams (including the penicillins and cephalosporins), aminoglycosides, lincosamides, tetracyclines, macrolides, quinolones, polypeptide antibiotics, and combinations of trimethoprim and sulfonamides (5, 6). The first choice antimicrobial agent for the treatment of *S. aureus* mastitis is penicillin G and in the case of beta-lactamase-producing and therefore penicillin-resistant strains, macrolides such as tylosin or isoxazolylpenicillins such as cloxacillin (5, 6). The growing prevalence of antibiotic resistant bacteria leading to treatment failures is a major health problem (7, 8). Therefore there is an increasing need for alternative methods such as hygiene optimization, the improvement of animal health using feed additives and the use of vaccines to manage infectious diseases in livestock (7). On dairy farms proper disinfection of milking equipment before and after the milking procedure remains crucial to avoid the spreading of mastitis pathogens from cow to cow. Such biocides must have broad-spectra activity against bacteria, but they should not adversely affect the milking equipment (9). Examples of antimicrobial agents used for these purposes include iodine-based products, which are most often used as an antiseptic for teat disinfection (10), but also various products containing phenolics, alcohol or chlorhexidine as germicides (11). However, it has been reported that in addition to antibiotic resistance, bacteria are less susceptible to commonly used antiseptics, e.g., chlorhexidine, benzalkonium chloride or povidone-iodine (12). Additionally, a high prevalence of genes responsible for benzalkonium chloride- and chlorhexidine-resistances has also been discovered in human *S. aureus* isolates (13). Thus, novel antimicrobial substances are urgently required (8). Historically natural sources such as soil microorganisms (bacteria, fungi) have been of special importance for the development of novel antimicrobials (14–17). However in recent years an increasing number of studies have been reporting the discovery of plant-derived antimicrobials (18). Recently benzaldehydes, isolated from essential oils from plants (e.g., almond oil), have shown antimicrobial activity against bacterial pathogens (16, 19–21). From all the tested benzaldehydesthe phenolic derivatives 2,3-dihydroxybenzaldehyde and 2,5-dihydroxybenzaldehyde (gentisaldehyde) have shown the highest antimicrobial activities (16).

In this study we investigated the antimicrobial activities of these two phenolic benzaldehydes using 172 bovine mastitis *S. aureus* isolates. These strains were characterized by *spa*-typing, antibiotic susceptibility testing and determination of minimum inhibitory concentrations (MICs) for three commonly used antiseptics (benzalkonium chloride, chlorhexidine and iodine solution). In addition to MIC determinations, we investigated the cytotoxicity of these phenolic benzaldehydes using bovine mammary epithelial MAC-T cells to evaluate their potential for use in the prevention of bovine mastitis.

## Materials and methods

### *S. aureus* isolates and cultivation

*S. aureus* strains (*n* = 172) from quarter milk samples of Austrian dairy cows with clinical or subclinical mastitis were isolated by the Upper Austrian Veterinary Health Service laboratory in Ried im Innkreis, Austria. Bacterial cultures were stored using cryogenic vials (Cryobank, CRYO/M, MAST GROUP Ltd., Bootle, United Kingdom). *Staphylococcus aureus* subsp. *aureus* ATCC® 29213™ (LGC Standards GmbH - Germany Office, Wesel, Germany) was used as a reference strain.

Bacteria were cultivated overnight on COS agar (Columbia-Agar supplemented with 5% sheep blood, Biomerieux, Marcy-l'Étoile, France) at 37°C. One colony was selected for all further investigations.

### Species identification and genotyping (*spa*-typing)

DNA isolation was performed using the Nucleospin® DNA-Extraction Kit (NucleoSpin® Tissue, Macherey-Nagel, Düren, Germany), according to the manufacturer's instructions. For species confirmation we used a modified species-specific PCR based on amplification of the *nuc*-gene, according to Brakstad et al. (22). The final reaction volume of 25 μl contained the following: 0.2 pmol/μl of each primer, 1X PCR buffer, 1.5 mM MgCl_2_, 2 U Platinum Taq DNA polymerase (Invitrogen™ Platinum™ Taq DNA Polymerase (kit), Carlsbad, USA), 0.2 mM dNTP-mix (Thermo Fisher Scientific, Waltham, USA), 5 μl of isolated genomic DNA and DEPC-treated water (Thermo Fisher Scientific). PCR cycling conditions were as follows: initial denaturation for 2 min at 94°C; followed by 30 cycles of denaturation at 94°C for 30 s, annealing at 55°C for 30 s and elongation at 72°C for 30 s; and final elongation at 72°C for 3.5 min. A negative (no DNA added) and a positive control (genomic DNA of *S. aureus* ATCC® 29213™) were included in each experiment. To detect genotype diversity, *spa*-typing was performed using the primers *spa*-1113F and *spa*-1514R (23). The final reaction volume of 50 μl included 0.18 pmol/μl of each primer, 1X PCR buffer, 3 mM MgCl_2_, 1.5 U Platinum Taq DNA polymerase, 0.4 mM dNTP-mix, 2 μl of isolated genomic DNA and DEPC-treated water. PCR cycling conditions were as follows: initial denaturation for 15 min at 94°C; followed by 30 cycles of denaturation at 94°C for 30 s, annealing at 59°C for 30 s and elongation at 72°C for 1 min; and a final elongation at 72°C for 10 min. Negative and positive controls were included similar to the *nuc*-PCR. The PCR products were sequenced (LGC Genomics GmbH, Berlin, Germany; Sanger sequencing) and *spa*-types determined using the ST*spa*TYPER website^1^ The sequence of novel *spa*-types was submitted to the RidomSpaServer^2^ for registration.

### Antibiotic susceptibilities

Antibiotic susceptibilities to eight antimicrobial agents (benzylpenicillin, cefoxitin, clindamycin, erythromycin, gentamicin, norfloxacin, tetracycline, and trimethoprim/sulfamethoxazole; Oxoid™ antibiotic discs, Thermo Fisher Scientific) belonging to eight different antimicrobial classes were determined using the disk diffusion method, according to EUCAST (24, 25). Antibiotic multi-resistance was defined as resistance to three or more antibiotic classes.

### MIC determinations

For MIC determination we used benzalkonium chloride (alkyl distribution from C_8_H_17_ to C_16_H_33_, Acros Organics, Geel, Belgium), chlorhexidine diacetate hydrate (Acros Organics), Lugol's solution [containing 5% (w/w) iodine and 10% (w/w) potassium iodide (both Merck, Darmstadt, Germany)], gentisaldehyde (2,5-dihydroxybenzaldehyde, Sigma-Aldrich, St. Louis, USA) and 2,3-dihydroxybenzaldehyde (Acros Organics). Stock solutions were freshly prepared in double-distilled water and sterile filtered using a 0.2 μm syringe filter before each experiment at the following concentrations: 3.2 × 10^4^ mg/L for benzalkonium chloride, 1.6 × 10^4^ mg/L for chlorhexidine, 5 × 10^4^ mg/L for iodine, 2 × 10^4^ mg/L for gentisaldehyde, and 5 × 10^3^ mg/L for 2,3-dihydroxybenzaldehyde. For MIC determination we used the following concentrations: 0.5, 1, 2, 4, and 8 mg/L for benzalkonium chloride and chlorhexidine; 62.5, 125, 250, 500, and 1,000 mg/L for iodine and 125, 250, 500, 1,000, and 2,000 mg/L for gentisaldehyde and 2,3-dihydroxybenzaldehyde.

For the MIC determination we used an automated robot-based microdilution method using the MicroLab Star Let pipetting robot (Hamilton Robotics, Reno, USA) and the automated Cytomat system, which included the Cytomat (Thermo Fisher Scientific), the Rack Runner (Hamilton Robotics) and the BioTek Reader (Synergy H1 Microplate Reader, BioTek Instruments, Inc., Winooski, USA).

Bacteria were initially cultivated in 1 ml of TSB (Tryptone Soya Broth, OXOID LTD., Basingstoke, England) for 6 h at 37°C. Afterwards 20 μl of bacterial culture was spotted onto tryptone soya agar (TSA, Trypto-Casein Soy Agar, Biokar Diagnostics, Beauvais, France) supplemented with 0.6% yeast extract (Biokar Diagnostics) prepared in a 96-well plate and incubated overnight at 37°C. Thereafter bacteria were inoculated in 200 μl TSB and incubated at 37°C for 8 h (stationary growth phase). Twenty microliter of bacterial culture was then inoculated into 1,380 μl of Mueller-Hinton broth (OXOID LTD, deep well plate) and incubated at 37°C for 18 h (stationary growth phase).The 96-well microtiter plates were prefilled with 100 μl Mueller-Hinton broth containing the antimicrobial compound, 80 μl Mueller-Hinton broth and 20 μl of the liquid bacterial cultures per well using the MicroLab Star Let pipetting robot. Each plate included eight wells of Mueller-Hinton broth with the antimicrobial compound at the specific concentration without inoculum. Additionally, bacteria were grown in Mueller-Hinton broth without any antimicrobial compound. Plates were sealed with sterile tape (NUNC, Thermo Fisher Scientific) and incubated in the automated Cytomat system at 37°C for 24 h. Absorbance was determined at 600 nm at 1 h intervals for 24 h. In total, three biological replicates were performed. The minimal concentration at which bacterial growth was inhibited for 24 h was defined as the MIC. The mean MIC was calculated from three biological replicates using Microsoft Excel. The MIC_50_ and MIC_90_ values were defined as the lowest concentration of the agents at which 50 and 90% of the isolates were inhibited, respectively.

### Cytotoxicity

The cytotoxicity of gentisaldehyde and 2,3-dihydroxybenzaldehyde was investigated by determining the percentage of metabolically active cells using the XTT assay. The XTT assay is based on the activity of the mitochondrial dehydrogenase enzyme at reducing XTT [2,3-bis-(2-methoxy-4-nitro-5-sulfophenyl)-2H-tetrazolium-5-carboxanilide] to an azure formazan. The quantity of formazan produced is proportional to the number of living cells and can be measured optically at 450 nm (26–28). Bovine mammary epithelial MAC-T cells were cultivated in Minimum Essential Medium with Earle's Balanced Salts (MEM/EBSS, GE Healthcare Life Sciences, HyClone Laboratories, South Logan, Utah, USA) supplemented with 10% fetal bovine serum (Gibco, Thermo Fisher Scientific), penicillin G (100 U/ml; AppliChem GmbH, Darmstadt, Germany), streptomycin sulfate (100 μg/ml, Fisher BioReagents™, Thermo Fisher Scientific), 5 μg/ml insulin (10 mg/mL in 25 mM HEPES, pH 8.2; Sigma-Aldrich), and 5 μg/ml hydrocortisone (Sigma-Aldrich) at 37°C in a humidified atmosphere (95% relative humidity) containing 5% CO_2_. For the cytotoxicity assay, 3 × 10^4^ cells per well were seeded in a 96-well microtiter plate and incubated overnight at 37°C. Cells were incubated for 24 h at 37°C in 200 μl MEM/EBSS with gentisaldehyde and 2,3-dihydroxybenzaldehyde at the following concentrations: 125, 250, 500, 1,000, and 2,000 mg/L. Cells incubated in media alone were used as a control and defined as 100% metabolically active cells. The cell supernatant was removed and cells washed four times with 100 μl Dulbecco's Phosphate-Buffered Saline (Gibco™, Thermo Fisher Scientific). Cells were incubated for 2 h at 37°C in 100 μl of MEM/EBBS and 25 μl 1 mg/ml XTT (Molecular Probes™ XTT, Invitrogen) solution prepared in MEM/EBBS supplemented with 1 μM phenanzinemethosulfate (PMS, Fisher Scientific). Absorbance was measured at 450 nm using the Tecan Instruments® reader before and shortly after the addition of the XTT solution and after two h of incubation at 37°C (Tecan Group Ltd., Männedorf, Switzerland). The mean values and standard deviation of four biological replicates performed in triplicate were calculated.

### Statistics

Microsoft® Excel 2007 and SPSS.20 software (SPSS Inc., Chicago USA) were used for statistical analysis. To determine significant associations between the MICs of the different antimicrobials, *spa*-type and antibiotic resistance (non-parametric values) we calculated the Phi-coefficient (*p* < 0.05). Phi-coefficient values (mathematical quantity) from 0.7 to 1 were defined as strong association, from 0.3 to 0.7 as weak and < 0.3 as little or no association. Only *spa*-types and antibiotic resistance shown by at least three strains were included. To determine correlations between the MIC of the different antimicrobial compounds, we used the Pearson correlation (*p* < 0.05). Pearson correlation values (mathematical amount) > 0.5 were defined as strong correlations, from 0.3 to 0.5 as moderate and from 0.1 to 0.3 as weak correlations. To analyze the cell viability data, we first used the Welch test to confirm variance homogeneity. Since data showed variance inhomogeneity, we used the *post-hoc* Games-Howell test to determine significant differences between cell viabilities (percentages of metabolically active cells) under different conditions (control, different concentrations of the test compounds); *p* < 0.05 were considered to be statistically significant.

## Results

### Genetic diversity and antibiotic resistance of bovine mastitis *S. aureus* strains

The 172 *S. aureus* strains belonged to 52 different *spa*-types, including five novel *spa*-genotypes (t16183, t16193, t16194, t16195, and t16196). The most prevalent *spa*-types were t529 (22% of isolates), t524 (11%), and t2953 (10%). Thirty-Eight *spa*-types (73%) were represented by less than three strains.

**Figure 1:**
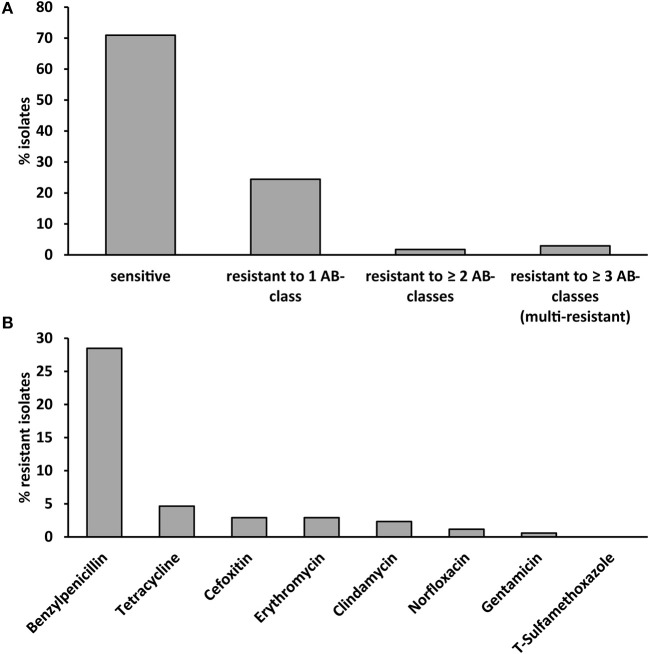
Antibiotic susceptibility of the 172 investigated *S. aureus* strains isolated from cases of bovine mastitis. **(A)** Antibiotic susceptibility, resistance and multi-resistance (resistance to ≥ 3 antibiotic classes). **(B)** Resistance to eight different antibiotics belonging to eight different antibiotic classes. T-sulfamethoxazole: trimethoprim/sulfamethoxazole.

Antibiotic susceptibility testing revealed that 24% of isolates were resistant to one, 2% to two antibiotic agents and 3% of isolates were multi-resistant (resistant to antibiotics belonging to three different classes, Figure 1A). Benzylpenicillin-resistance was the most common resistance (29% of isolates) followed by tetracycline-resistance (5% of isolates, Figure 1B). Less than 3% of strains were resistant to other antibiotic agents. None of the *S. aureus* strains were resistant to trimethoprim/sulfamethoxazole.

The occurrence of any type of antibiotic resistance correlated with the *spa*-type (phi coefficient 0.860, *p* < 0.001, Table S1). We observed a strong association between the *spa* type and antibiotic resistance for all antibiotic classes (phi coefficient 0.738–1, *p* < 0.001) except gentamycin (phi coefficient 0.573, *p* < 0.001, Table S1). All strains of t024 (*n* = 6) and t091 (*n* = 3), 72% of t2953 strains (*n* = 18) and 60% of t008 (*n* = 5) strains were resistant to benzylpenicillin (Table S2). All three t001 strains were resistant to benzylpenicillin, cefoxitin, and tetracycline.

### Benzalkonium chloride, chlorhexidine, and iodine susceptibilities

**Figure 2:**
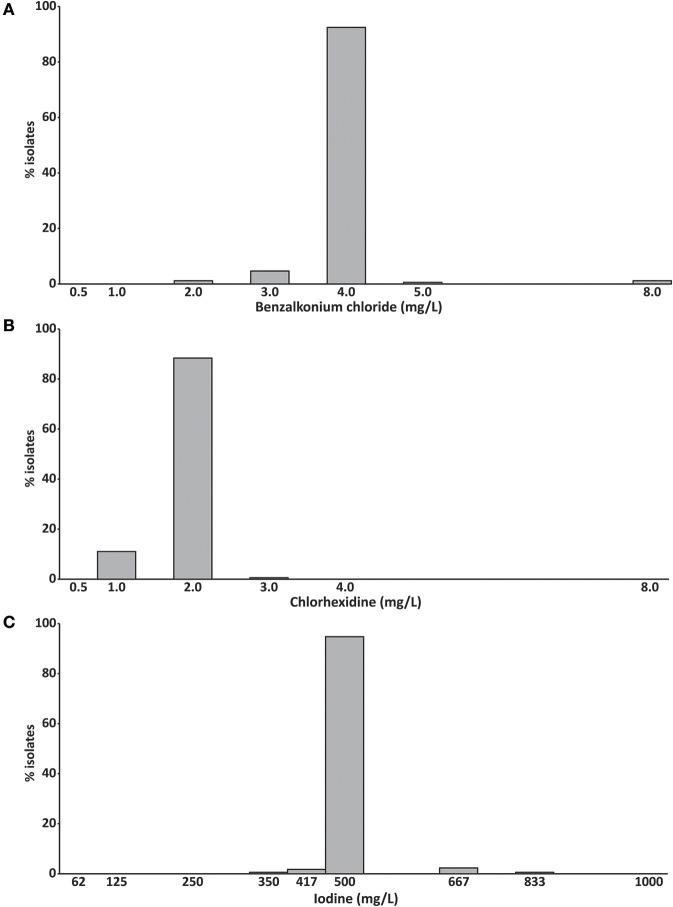
Minimum inhibitory concentrations of **(A)** benzalkonium chloride, **(B)** chlorhexidine and **(C)** iodine (mg/L) of 172 bovine mastitis *S. aureus* isolates. Data are presented as mean MICs of three independent replicates.

Benzalkonium chloride inhibited the growth of 92% of *S. aureus* isolates at a concentration of 4 mg/L and chlorhexidine inhibited the growth of 88% of isolates at 2 mg/L (Figures 2A,B). The growth of 95% of isolates was inhibited by 500 mg/L iodine (Figure 2C). No difference between the MIC_50_ and MIC_90_ was observed for all three compounds (Table 1).

**Table 1:** Minimum inhibitory concentrations (MIC) of 172 bovine mastitis *S. aureus* isolates.

	**Range-tested (mg/L)**	**Range (mg/L)**	**MIC_50_**	**MIC_90_**
Benzalkonium chloride	0.5-8	2-8	4	4
Chlorhexidine	0.5-8	1-3	2	2
Iodine	62.5-1,000	350-833	500	500
Gentisaldehyde	125-2,000	500-1,000	500	1,000
2,3-Dihydroxybenzaldehyde	125-2,000	500-1,000	500	833

*The MIC_50_ and MIC_90_ were defined as the lowest concentrations at which 50 and 90% of the isolates were inhibited, respectively*.

We observed a weak correlation between the MICs of benzalkonium chloride and chlorhexidine (Pearson correlation *R* = 0.191, *p* = 0.012, Table S3), the *spa*-type and the MIC of benzalkonium chloride (phi coefficient 0.633, *p* = 0.002), and the *spa*-type and the MIC of chlorhexidine (phi coefficient, 0.579, *p* < 0.001, Table S4). The benzalkonium chloride MIC also correlated also with the occurrences of cefoxitin and tetracycline resistance (phi coefficient 0.306 and 0.251, *p* < 0.03). Interestingly, the chlorhexidine MIC correlated significantly with the occurrences of all different antibiotic resistances except benzylpenicillin (phi coefficients: 0.217–0.536, *p* < 0.02). No correlation between the iodine MIC, the *spa*-type and any antibiotic resistance was detected.

### Antimicrobial activities of gentisaldehyde and 2,3-dihydroxybenzaldehyde

**Figure 3:**
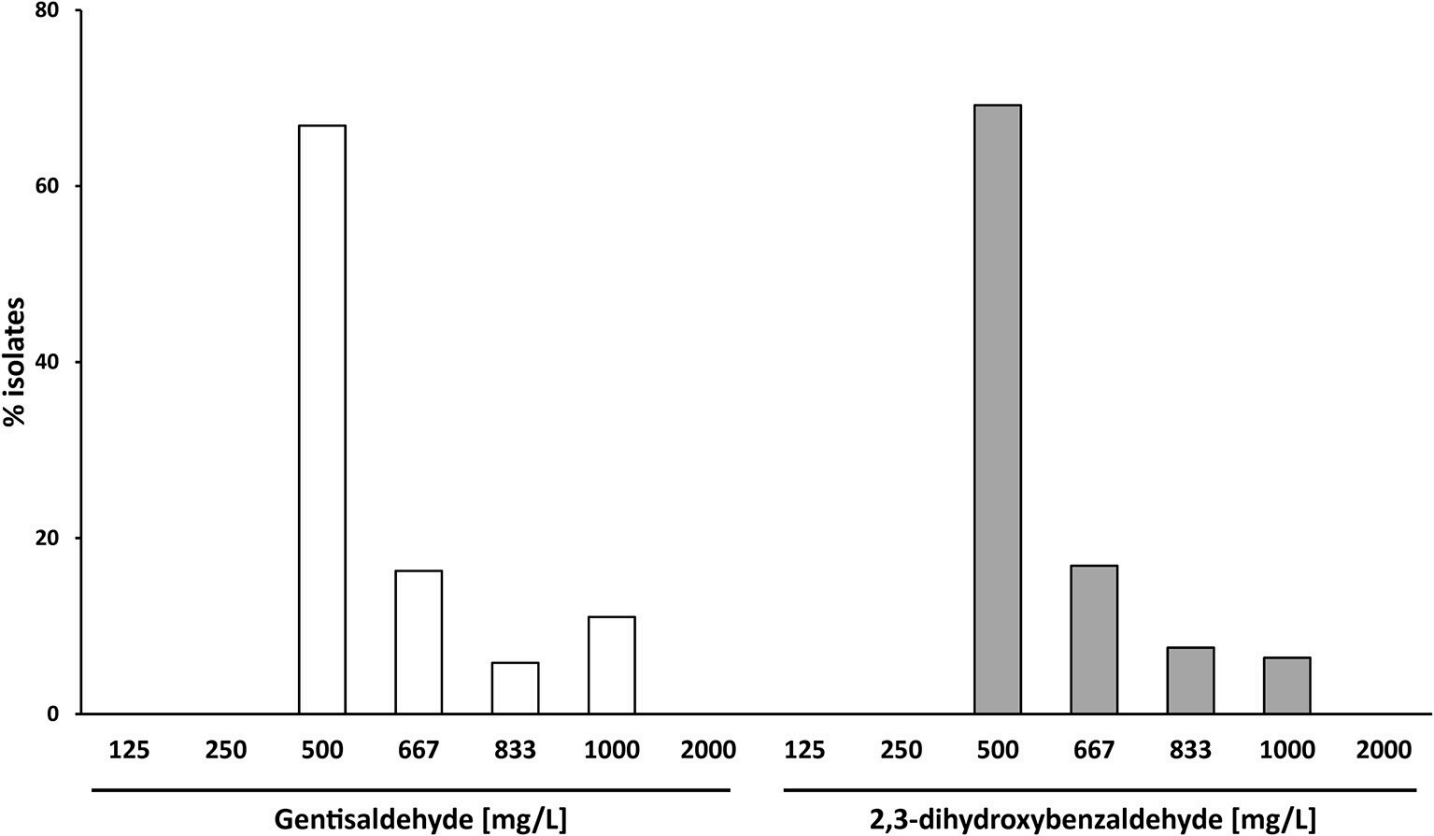
Minimum inhibitory concentrations of gentisaldehyde and 2,3-dihydroxybenzaldehyde (mg/L) of 172 bovine mastitis *S. aureus* isolates. Data are presented as mean MICs of three independent replicates.

The MIC_50_ of both compounds was 500 mg/L (Table 1). Sixty-Seven percent of *S. aureus* isolates showed a MIC of 500 mg/L for gentisaldehyde and 69% for 2,3-dihydroxybenzaldehyde, respectively (Figure 3). No *S. aureus* strains showed a MIC lower than 500 mg/L and higher than 1,000 mg/L for both compounds. The MIC_90_ was 1,000 mg/L for gentisaldehyde and 833 mg/L for 2,3-dihydroxybenzaldehyde (Table 1).

We observed a moderate correlation between the MIC of gentisaldehyde and 2,3-dihydroxybenzaldehyde (Pearson correlation *R* = 0.497, *p* < 0.0001, Table S3). The gentisaldehyde MIC correlated weakly with the occurrences of gentamicin and erythromycin resistances (phi coefficient 0.308 and 0.262, *p* < 0.01), whereas the MIC of 2,3-dihydroxybenzaldehyde was associated with the *spa*-type (phi-coefficient 0.695, *p* = 0.013) and the occurrence of resistance to all antibiotic classes except tetracycline (phi-coefficient 0.222–0.376, *p* < 0.05, Table S4).

### Cytotoxicity

**Figure 4:**
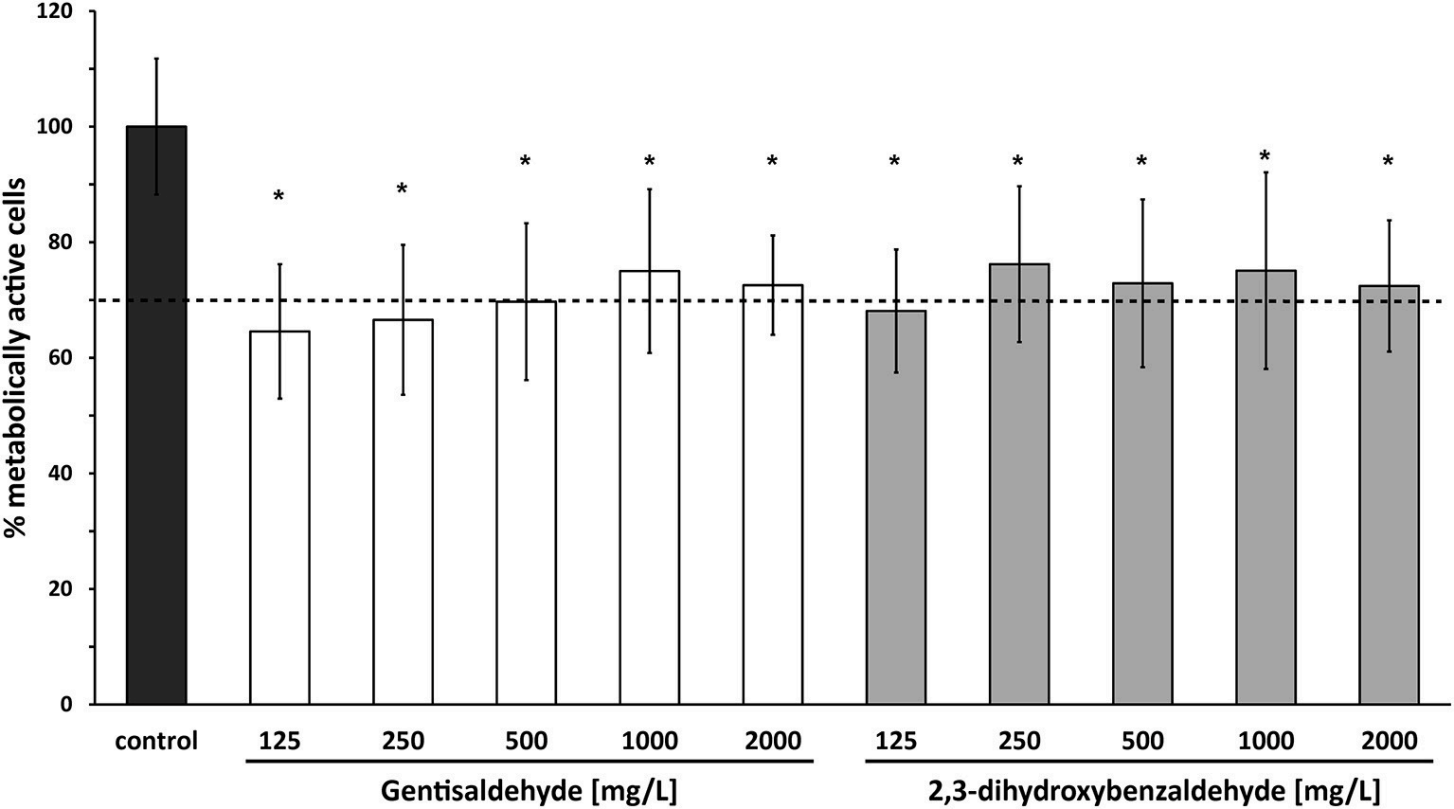
Cytotoxicity of gentisaldehyde and 2,3-dihydroxybenzaldehyde. Metabolically active bovine mammary epithelial MAC-T cells (%) were measured after 24 h of treatment with gentisaldehyde and 2,3-dihydroxybenzaldehyde (125–2,000 mg/L). Control: cells incubated in media without any compound. Data represent mean values ± SD of four biological replicates performed in triplicate. ^*^p < 0.05 indicates a statistically significant difference compared to the control. Dashed line indicates threshold of 70% metabolically active cells, defined as non-toxic.

Cytotoxicity of both compounds at all tested concentrations in bovine mammary epithelial MAC-T cells was low. The percentage of metabolically active cells was higher than 64% for all tested gentisaldehyde and 2,3-dihydroxybenzaldehyde concentrations (Figure 4). However, cell viability was significant lower at all concentrations compared to the control. No significant difference between cell viabilities at MIC_50_ and MIC_90_ was observed for both compounds.

## Discussion

The increasing rate of bacteria resistant to antibiotics and antiseptics poses a major problem to human and veterinary health. Consequently, there is a desperate demand for novel antimicrobial agents active against resistant bacteria (8, 12, 29). In recognition of this requirement, this study investigated alternative antimicrobial compounds that might be suitable for the prevention of bovine mastitis. We examined two promising phenolic benzaldehydes reported to have antibacterial activities (16, 21).

The bovine *S. aureus* mastitis strain set used in this study showed a high number of different *spa*-types, indicating high genotype diversity. The most prevalent *spa*-types have also been found with a similar occurrence in other studies that have investigated bovine *S. aureus* isolates (30–32). The strain set comprised 24% of isolates resistant to one antibiotic class and a low number of multi-resistant strains. Most commonly observed was resistance to benzylpenicillin (29%) and tetracycline (5% of isolates), whereas there were no isolates resistant to the trimethoprim/sulfamethoxazole combination. A recent study showed a similar prevalence of penicillin resistant *S. aureus* strains isolated in Switzerland and France (33). Further, low or even no resistance to the sulfatrimethoprim combination has also been reported in other countries (34). Multi-resistance of *S. aureus* in bovine mastitis has been reported for 1.4–17% of isolates. However, mono- and multi-resistance of *S. aureus* mastitis isolates varies highly across different countries (34). Further, as the term “multi-resistance” is often not clearly defined, it is difficult to make direct comparison between different studies and countries.

The occurrence and the type of antibiotic resistance strongly correlated with the *spa-*type. Recently, this has also been reported between clindamycin and erythromycin resistances and the *spa*-type; although there were no correlations between the occurrences of cefoxitin, gentamicin, tetracycline, and trimethoprim/sulfamethoxazole resistances (35). However, in this study the *spa*-types have been identified by PCR and not by sequencing. In respect of bovine *S. aureus* isolates, *spa*-types t2953 and t524 have been reported to be penicillin resistant (33). Similarly, 72% of *S. aureus* strains belonging to genotype t2953 showed penicillin resistance in our study, whereas only one isolate of *spa*-type t524 was penicillin resistant. Several *spa*-types, such as t011, which was also found in this study, are known to be methicillin- and multi-resistant (36).

Data on the susceptibility of *S. aureus* mastitis strains to antiseptics are rare. In the present study we found susceptibility to three antiseptics with MIC_50_ (and MIC_90_) values of 4 mg/L for benzalkonium chloride, 2 mg/L for chlorhexidine and 500 mg/L for iodine. A recent study including 37 *S. aureus* mastitis strains reported higher MIC_50_ and MIC_90_ values for iodine compared to our study, but a similar MIC for chlorhexidine (37).

A weak correlation between the MICs of benzalkonium chloride and chlorhexidine was found, which suggests a similar mode of action. This mode of action appears to be primarily linked to modification and impairment of cell membrane permeability (38, 39). Consequently, the presence of efflux pumps in bacteria can especially confer higher tolerance to both compounds (40, 41). Since the occurrence of multidrug efflux pumps such as NorA, NorB or MepA results in multiple antimicrobial resistance (41), we expected a strong correlation between benzalkonium chloride and chlorhexidine MICs and antibiotic resistance. However, we detected only a weak association, mainly between the chlorhexidine MIC and the occurrence of antibiotic resistance (except to benzylpenicillin). The reason could be the low number of resistant strains included in this study. Alternatively, the MIC for iodine did not correlate with any other parameter. This is in line with other studies showing almost no cross-resistance between iodine and other antibiotics in bovine mastitis *S. aureus* (37, 42). The exact mode of action of iodine is still not clear. However, iodine is known to affect rapidly key groups of proteins, nucleotides and fatty acids (43).

Both test compounds—gentisaldehyde as well as its structural derivate 2,3-dihydroxybenzaldehyde—revealed antimicrobial activities at low-toxic concentrations. The MIC_50_ values of both compounds were high (500 mg/L), equivalent to that of iodine. Friedman et al. revealed similar antimicrobial activities for the two dihydroxybenzaldehydes against *Campylobacter jejuni, Escherichia coli, Listeria monocytogenes*, and *Salmonella enterica* using only one strain of each species (16). Their measured BA_50_ (bacterial activity) values for all four pathogens were 1,460 mg/L for gentisaldehyde and 670 mg/L for 2,3-dihydroxybenzaldehyde.

We revealed a moderate correlation between the MIC values for both compounds. The MIC value of both compounds also correlated with the *spa*-type at similar levels, but this correlation was only significant for 2,3-dihydroxybenzaldehyde. We also detected differences between the two compounds in respect of correlations between their MICs and the occurrence of antibiotic resistance. However, since the correlations were weak and only a limited number of resistant isolates were included in this study, it is difficult to speculate whether gentisaldehyde has higher antimicrobial activity against antibiotic resistant strains than 2,3-dihydroxybenzaldehyde. Since we observed no correlation between the MIC of the antiseptics and the two new compounds, we can conclude that *S. aureus* strains resistant to common antiseptics are likely susceptible to gentisaldehyde and 2,3-dihydroxybenzaldehyde. Furthermore, this suggests a different mode of action and resistance mechanism. Nevertheless, the mode of action of gentisaldehyde and 2,3-dihydroxybenzaldehyde has still to be elucidated.

Results of the cytotoxicity testing of the two benzaldehydes underscores their potential use as antiseptics, since more than 64% of cells were viable after incubation with the compounds at concentrations ranging from 125 to 2,000 mg/L. A threshold of more than 70% viable cells is conventionally defined as non-toxic (27, 28, 44), and therefore gentisaldehyde and 2,3-dihydroxybenzaldehyde show low toxicity to bovine mammary epithelial cells. The cytotoxicity of these compounds has not been tested before. Since bovine mammary cells are not the ideal model for teat disinfectants further cytotoxicity testing e.g. using primary bovine skin cells are needed.

In conclusion, we show here that gentisaldehyde and its structural derivate 2,3-dihydroxybenzaldehyde have antimicrobial activities on a diverse set of bovine *S. aureus* mastitis isolates, including (multi-) resistant strains. As both compounds show low toxicity to bovine mammary cells they are potential candidates for further investigation as alternative antimicrobials in the prevention of bovine mastitis. Further studies to examine their antimicrobial activities against other common mastitis pathogens (e.g., streptococci and Gram-negative species) and elucidate their mode of action are warranted.

## Data availability statement

Data generated during the presented study are available from the corresponding author (KR) upon reasonable request.

## Author contributions

Isolates were collected and provided by BL. Methods were developed by AS, CZ, and KR. All experiments were performed by AS. Raw data were evaluated by AS. Statistical analyses were performed by KR. Results were interpreted by AS and KR. KR and MW coordinated the study. All authors read and approved the final manuscript.

### Conflict of interest statement

The authors declare that the research was conducted in the absence of any commercial or financial relationships that could be construed as a potential conflict of interest.

## Footnotes

^1^ST *spa*TYPER, URL: http://spatyper.fortinbras.us/

^2^RidomSpaServer of the Ridom GmbH, URL: http://spa.ridom.de/
