# Supplementary material for: Gentisaldehyde and Its Derivative 2,3-Dihydroxybenzaldehyde Show Antimicrobial Activities Against Bovine Mastitis Staphylococcus aureus
Source: Front Vet Sci. 2018 Jul 11;5:148. doi: 10.3389/fvets.2018.00148 (PMC6050399; doi:10.3389/fvets.2018.00148)
Supplement: Supplementary file 1 [file Data_Sheet_1.pdf]

Table S1 Associations between the presence of antibiotic resistance and the *spa*-type.

	***spa*-type**	
	value	sig.
Antibiotica resistance	0.860	<0.001
Benzylpenicillin	0.846	<0.001
Cefoxitin	1.000	<0.001
Gentamicin	0.573	<0.001
Tetracycline	0.798	<0.001
Clindamycin	0.813	<0.001
Erythromycin	0.738	<0.001

Value: Phi coefficient; mathematical value of the Phi coefficient 0-0.3: little to no association, 0.3-0.7: weak association, 0.7-1: strong association; sig. = approximated significance;

Table S2: Antibiotic resistance of the most prevalent *spa*-types (n≥3)

***spa*-type**	**number of isolates**	**susceptible (%)**	**resistant (%)**							
			**BP**	**FOX**	**SXT**	**CN**	**NOR**	**TET**	**DA**	**ERY**
**t529**	38	100	0	0	0	0	0	0	0	0
**t2953**	18	28	72	0	0	0	0	0	0	0
**t524**	17	94	6	0	0	0	0	6	0	0
**t3380**	8	100	0	0	0	0	0	0	0	0
**t024**	6	0	100	0	0	0	0	0	0	0
**t13487**	6	100	0	0	0	0	0	0	0	0
**t899**	6	100	0	0	0	0	0	0	0	0
**t008**	5	20	60	0	0	0	0	20	0	0
**t16183**	5	100	0	0	0	0	0	0	0	0
**t2873**	5	100	0	0	0	0	0	0	0	0
**t521**	4	75	25	0	0	0	0	0	0	0
**t011**	3	0	100	100	0	33	0	100	67	67
**t091**	3	0	100	0	0	0	0	0	0	33
**t528**	3	100	0	0	0	0	0	0	0	0

R: resistant, BP: benzylpenicillin, FOX:cefoxitin, SXT: trimethoprim/sulfamethoxazole, CN: gentamicin, NOR: norfloxacin, TET: tetracycline, DA: clindamycin, ERY: erythromycin

Table S3 Pearson correlation between the MICs of benzalkonium chloride, chlorhexidine, iodine, gentisaldehyde and 2,3-dihydroxybenzaldehyde (2,3-DHBA).

	**Chlorhexidine**	**Iodine**	**Gentisaldehyde**	**2,3-DHBA**
**Benzalkonium chloride**				
Pearson correlation	**0.191**	0.004	-0.067	0.007
Significance	**0.012**	0.959	0.386	0.932
**Chlorhexidine**				
Pearson correlation	1.000	0.077	-0.071	-0.016
Significance		0.318	0.353	0.837
**Iodine**				
Pearson correlation		1.000	-0.001	-0.070
Significance			0.987	0.360
**Gentisaldehyde**				
Pearson correlation			1.000	**0.497**
Significance				**<0.001**

0.1 $<|r|<0.3$: weak correlation; 0.3 $<|r|<0.5$: moderate correlation; $|r|>0.5$: strong correlation; bold: significant correlation $p<0.05$

Table S4 Associations between the MICs of the different antimicrobials, the presence of antibiotic resistance and the *spa*-type.

	**Gentisaldehyde**		**2,3-Dihydroxy-benzaldehyde**		**Benzalkonium chloride**		**Chlorhexidine**		**Iodine**	
	value	sig.	value	sig.	value	sig.	value	sig.	value	sig.
spa-type	0.650	0.059	**0.695**	**0.013**	**0.633**	**0.002**	**0.579**	**<0.001**	0.623	0.125
Antibiotica resistance	0.098	0.647	0.212	0.051	0.153	0.405	0.159	0.155	0.147	0.294
Benzylpenicillin	0.107	0.576	**0.222**	**0.037**	0.149	0.432	0.139	0.189	0.150	0.274
Cefoxitin	0.119	0.488	**0.284**	**0.003**	**0.306**	**0.003**	**0.474**	**<0.001**	0.043	0.957
Gentamicin	**0.308**	**0.001**	**0.267**	**0.006**	0.022	0.999	**0.217**	**0.017**	0.019	0.996
Tetracycline	0.100	0.631	0.177	0.144	**0.251**	**0.029**	**0.398**	**<0.001**	0.055	0.915
Clindamycin	0.148	0.289	**0.300**	**0.001**	0.044	0.987	**0.536**	**<0.001**	0.038	0.969
Erythromycin	**0.262**	**0.008**	**0.376**	**<0.001**	0.049	0.981	**0.474**	**<0.001**	0.043	0.957

value= Phi coefficient; mathematical value of the Phi coefficient 0-0.3: little to no association, 0.3-0.7: weak association, 0.7-1: strong association; sig. = approximated significance; bold: significant associations (p<0.05)
